# Three-dimensional genomic structure and aroma formation in the tea cultivar ‘Qiancha 1’

**DOI:** 10.1093/hr/uhaf064

**Published:** 2025-02-26

**Authors:** Dahe Qiao, Wenlong Lei, Xiaozeng Mi, Chun Yang, Sihui Liang, Huike Li, Yan Guo, Juan Chen, Wenmin Fan, Jiawei Yan, Wen Yang, Wenjia Zheng, Taiming Jiang, Youben Yu, Zhengwu Chen, Pengjie Wang

**Affiliations:** Guizhou Tea Research Institute, Guizhou Academy of Agricultural Sciences, Guiyang, Guizhou 550006, China; Key Laboratory of Crop Genetic Resources and Germplasm Innovation in Karst Region, Ministry of Agriculture and Rural Affairs, Guiyang, Guizhou 550006, China; College of Horticulture, Northwest A&F University, Xianyang 712199, China; Guizhou Tea Research Institute, Guizhou Academy of Agricultural Sciences, Guiyang, Guizhou 550006, China; Guizhou Tea Research Institute, Guizhou Academy of Agricultural Sciences, Guiyang, Guizhou 550006, China; Guizhou Tea Research Institute, Guizhou Academy of Agricultural Sciences, Guiyang, Guizhou 550006, China; College of Horticulture, Northwest A&F University, Xianyang 712199, China; Guizhou Tea Research Institute, Guizhou Academy of Agricultural Sciences, Guiyang, Guizhou 550006, China; Guizhou Tea Research Institute, Guizhou Academy of Agricultural Sciences, Guiyang, Guizhou 550006, China; College of Horticulture, Northwest A&F University, Xianyang 712199, China; College of Horticulture, Northwest A&F University, Xianyang 712199, China; Guizhou Academy of Agricultural Sciences, Guiyang, Guizhou 550006, China; Guizhou Tea Research Institute, Guizhou Academy of Agricultural Sciences, Guiyang, Guizhou 550006, China; Guizhou Academy of Agricultural Sciences, Guiyang, Guizhou 550006, China; College of Horticulture, Northwest A&F University, Xianyang 712199, China; Guizhou Tea Research Institute, Guizhou Academy of Agricultural Sciences, Guiyang, Guizhou 550006, China; College of Horticulture, Northwest A&F University, Xianyang 712199, China

Dear Editor,

Recent advances have provided preliminary insights into the 3D genome of tea plants [1]; structural variations and transposable elements have been shown to significantly influence gene expression and phenotypic traits across various species, including tea plants [2], apple, and tomato. However, significant gaps remain in understanding the relationship between structural variations, transposable elements, and the 3D genome structure. ‘Qiancha 1’ stands out among promoted green tea cultivars in China, renowned for its rich floral aroma, which is heavily influenced by the leaf withering process [3]. Despite its growing popularity and cultivation across over 3000 hectares in various provinces of China, the formation and regulatory mechanisms of this intense floral aroma, as well as its association with 3D genome organization, have yet to be thoroughly explored.

A total of 135 Gb of PacBio HiFi reads were generated for the ‘Qiancha 1’ accession. The initial *de novo* genome assembly utilizing hifiasm resulted in 3.3 Gb of sequences. Using *k*-mer counting with Khaper, 200 Mb of heterozygous sequences were filtered, resulting in a 3.10 Gb assembly with a contig N50 of 132.6 Mb and achieving 99.2% BUSCO completeness for the ‘Qiancha 1’ genome. This is significantly higher than for other tea cultivars [2] (Fig. 1A). Chromosome-level genome assembly was achieved by anchoring contigs to 15 pseudochromosomes (Fig. 1B). Assembly assessment utilizing long terminal repeat (LTR) annotation showed that the LTR assembly index (LAI) was 11.4 (Fig. 1A), additionally, the Hi-C contact heat map and synteny analysis with the high-quality ‘Huangdan’ further demonstrated the enhanced continuity and completeness of the ‘Qiancha 1’ genome (Supplementary Data Figs S1 and S2).

**Figure 1:**
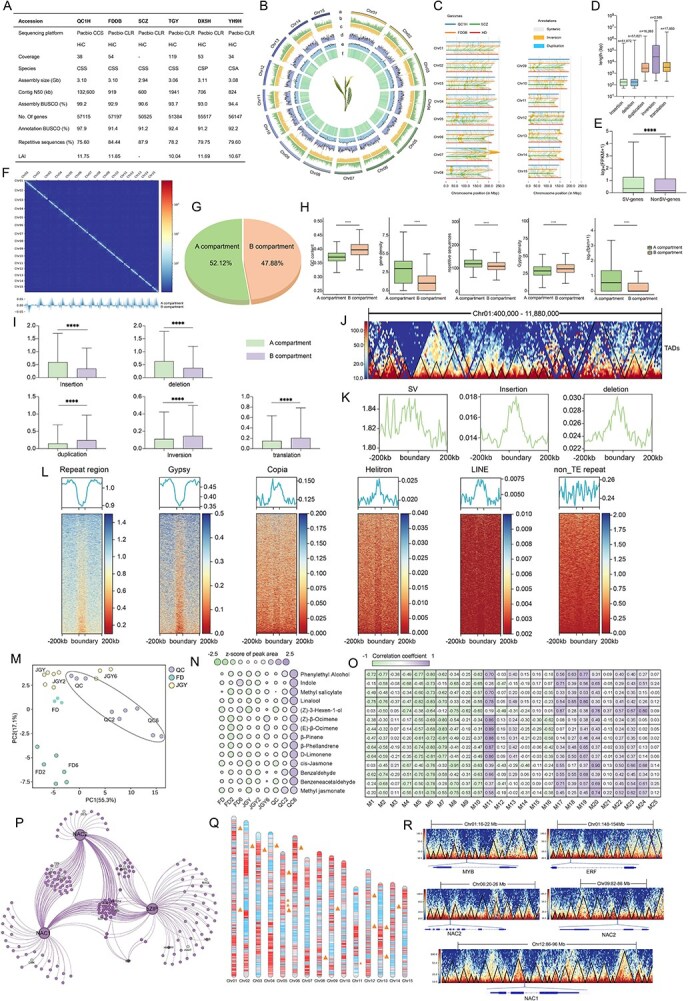
The genome and 3D chromatin of ‘Qiancha 1’. ^****^P < 0.0001 (Wilcoxon test). **A** Comparison of ‘Qiancha1’ with other tea plants. **B** Circos plot showing genome details. (a) Chromosome; (b) gene density; (c) TE density; (d) Copia density; (e) Gypsy density. **C** Comparison of SVs between ‘Qiancha 1’, ‘FDDB’, ‘SCZ’ and ‘HD’. **D** Boxplots of SV sizes. **E** Expression level comparisons between SV genes and non-SV genes. **F** Genome-wide Hi-C interaction matrix and A and B compartments. **G** Proportions of A and B compartments. **H** Genome features of A and B compartments. **I** Counts of A and B compartments with SVs. **J** Visualization of TAD structure from Chr01: 400 000–11 880 000 bp. **K** Distribution of SVs around TAD boundaries. **L** Enrichment of repetitive sequence around TAD boundaries. **M** PCA plot of volatile metabolites across three tea plant cultivars in fresh leaves after 2 and 6 hours of spreading. **N** Analysis of volatile metabolite from fresh leaves to spreading in three tea cultivars. **O** Heat map of the correlation between volatile metabolite and co-expression modules. **P** Transcriptional regulatory network of volatile metabolites in the M6, 11 and 20 modules. **Q** The distribution of the 18 co-expressed genes associated with aroma is localized within the A compartment. **R** Distribution of transcription factors across TADs.

We predicted 57 115 protein-coding genes through the GETA pipeline, collectively achieving 97.9% BUSCO completeness. Furthermore, a total of 2.35 Gb (75.60%) repetitive sequences in ‘Qiancha 1’ were annotated.

Extensive and diverse structural variations (SVs) are present in the tea plant genome, potentially influencing various traits such as leaf color and the formation of metabolic components [2]. To catalog the structural variations within the ‘Qiancha 1’ genome, a comparative analysis was performed against the green tea representative cultivars ‘Shuchazao’ (SCZ) and ‘Fuding Dabaicha’ (FDDB), as well as the oolong tea representative cultivar ‘Huangdan’ (HD) (Fig. 1C). A total of 139 393 SVs were identified, consisting of 51 072 insertions, 51 621 deletions, 16 265 duplications, 2585 inversions and 17 850 translocations (Fig. 1D). Moreover, comparison with ‘FDDB’, which serves as the standard reference for green tea cultivars in China, revealed that genes affected by SVs showed significantly higher expression levels compared with those not overlapping with SVs (Fig. 1E), suggesting a potential correlation between SVs and gene expression levels in tea plants.

3D chromatin organization can be arranged into compartments, topologically associating domains (TADs), and chromatin loops; they can bring distal *cis*-regulatory elements into spatial proximity with their target genes and play a pivotal role in gene regulation [4]. PCA of the Hi-C contact, assessed at 100-kb resolution, indicated that the A and B compartments account for 52.12% and 47.88%, respectively (Fig. 1F and G). Using 40-kb resolution Hi-C contact, 5064 TADs were identified (Fig. 1J). We subsequently examined the genomic features at different hierarchical levels of spatial structure in ‘Qiancha 1’, and our finding was consistent with prior research on the genomes of soybean [5]: the A compartment exhibited higher gene density, greater gene expression levels, and lower GC content compared with the B compartment (Fig. 1H). The distribution of SVs exhibited preferences within the A and B compartments. The ratios of insertions and deletions were significantly higher in the A compartment, while duplications, inversions, and translocations were preferentially distributed in the B compartment (Fig. 1I). Although the overall distribution of SVs at TAD boundaries did not exhibit a discernible pattern, insertions and deletions were significantly enriched at TAD boundaries (Fig. 1K).

Repetitive sequences constitute 75.60% of the ‘Qiancha 1’ genome, yet the relationship between repetitive elements and 3D chromatin architecture in ‘Qiancha 1’ remains unknown. Repetitive sequences were more abundant in the A compartment, yet the most prevalent Gypsy transposon exhibited an opposite trend (Fig. 1H). This pattern is not consistent with those observed in most plant 3D genomes reported thus far. We found that repetitive sequences were depleted at TAD boundaries; however, interestingly, there was significant enrichment of Copia, LINE, and Helitron transposons around TAD boundaries, except for Gypsy transposons, which were the most abundant in ‘Qiancha 1’ (Fig. 1L). In conclusion, the uneven distribution of structural variants and repetitive sequences may contribute to the formation of the 3D structure of the ‘Qiancha 1’ genome.

Furthermore, a non-supervised metrology PCA on the identified metabolites across three cultivars (‘Qiancha 1’, ‘FDDB’, ‘JGY’) showed that the volatile metabolites in various cultivars were clearly separated (Fig. 1M). Specific volatiles, including phenylethyl alcohol, indole, methyl salicylate, (*Z*)-*β*-ocimene, (*E*)-*β*-ocimene, and jasmone, were found to be significantly accumulated and were particularly prominent after 6 hours of withering in ‘Qiancha 1’ (Fig. 1N). These components may collectively contribute to the characteristic floral and fruity aroma profile of ‘Qiancha 1’. Weighted correlation network analysis (WGCNA) was performed on samples from three tea cultivars. Focusing on co-expression modules associated with volatile compounds in ‘Qiancha 1’, a total of 25 modules were identified (Fig. 1O). Ten genes potentially involved in the synthesis and regulation of volatile metabolites were identified, including 4-diphosphocytidyl-2-C-methyl-d-erythritol kinase (*CMK*), 4-hydroxy-3-methylbut-2-en-1-yl diphosphate synthase (*HMBPP*), and hydroxymethylbutenyl diphosphate reductase (*HDR*), all of which are involved in the MEP pathway, three terpene synthase genes (*TPS*), and (*S*)-8-oxocitronellyl enol synthase (*ISY1*), which is involved in the synthesis of terpene compounds, as well as aromatic amino acid aminotransferase 1 (*AAAT*) and ocimene synthase (*OCS*), which are located in M6, 11 and 20 modules; their expression was highly correlated with the accumulation of volatile metabolites. Furthermore, eight transcription factors (TFs), comprising members of the bZIP, NAC, MYB, and ERF families, which exhibited high correlation with the 10 genes involved in the synthesis and regulation of volatile metabolites, formed a correlation network (Fig. 1P). This suggests that these TFs may play a dominant role in floral and fruity aroma profile of ‘Qiancha 1’.

To elucidate the relationship between associated genes related to aroma and 3D genome architecture, we performed a spatial localization analysis of these genes. It was discovered that they are specifically localized to the A compartment (Fig. 1Q). Furthermore, the five TFs within the co-expression network were found to be enriched at the boundaries of TADs (Fig. 1R), which are regions crucial for the regulation of gene expression. These findings suggest that associated genes related to aroma may be subject to regulation at the level of 3D genome architecture.

In summary, the high-quality assembly of the ‘Qiancha 1’ genome provides a reliable reference for the analysis of 3D genome structure, aids in understanding the formation and regulatory mechanisms of the intense floral aroma during the dissemination process, and provides a robust foundation for further research.

## Data Availability

The data that support the findings of this study are publicly available in the National Genomics Data Center (https://ngdc.cncb.ac.cn/) under project number PRJCA028918.
